# Studying the Evolution of Neural Activation Patterns During Training of Feed-Forward ReLU Networks

**DOI:** 10.3389/frai.2021.642374

**Published:** 2021-12-23

**Authors:** David Hartmann, Daniel Franzen, Sebastian Brodehl

**Affiliations:** Visual Computing Group, Institute of Computer Science, Faculty of Physics, Mathematics and Computer Science, Johannes Gutenberg-University, Mainz, Germany

**Keywords:** activation patterns, neural activations, feed-forward networks, RELU, activation entropy

## Abstract

The ability of deep neural networks to form powerful emergent representations of complex statistical patterns in data is as remarkable as imperfectly understood. For deep ReLU networks, these are encoded in the mixed discrete–continuous structure of linear weight matrices and non-linear binary activations. Our article develops a new technique for instrumenting such networks to efficiently record activation statistics, such as information content (entropy) and similarity of patterns, in real-world training runs. We then study the evolution of activation patterns during training for networks of different architecture using different training and initialization strategies. As a result, we see characteristic- and general-related as well as architecture-related behavioral patterns: in particular, most architectures form bottom-up structure, with the exception of highly tuned state-of-the-art architectures and methods (PyramidNet and FixUp), where layers appear to converge more simultaneously. We also observe intermediate dips in entropy in conventional CNNs that are not visible in residual networks. A reference implementation is provided under a free license^1^.

## 1 Introduction

The increased attention that deep neural networks have received in the literature over the past decade is arguably due to the stark contrast between their technical simplicity and their high practical performance for complex statistical pattern modeling tasks. The actual computational structure responsible for their remarkable performance is an emergent property of the training dynamics rather than pre-engineered (as in many classical machine learning approaches). Early works on deep networks have already visualized the emerged structures (Zeiler and Fergus, 2014), and despite many remarkable observations (such as Frankle et al. (2020); Frankle and Carbin (2019); Achille et al. (2019); Gur-Ari et al. (2018), to name only a few), the literature still lacks a complete picture of how these structures emerge during training.

In contrast to purely linear classifiers, deep networks have a much higher expressive power, resulting from non-linear activation functions between linear maps. One such non-linearity, the ReLU activation (Nair and Hinton, 2010), is of particular interest for research on training dynamics. While ReLU-based networks have provided state-of-the-art results in many applications in the literature (He et al., 2015), and the computational structure of the ReLU non-linearity is very simple. As the non-linearity only has two states (zero or pass-through, “inactive” and “activated”), the expressive power of a fully trained network hinges upon the acquired ability to select suitable activations for different inputs.

In this work, we utilize the discrete decisions made inside ReLU networks to analyze the evolution of the expressivity of deep networks during training. In more detail, by measuring the distribution of activation patterns seen in the whole training data set, our article makes the following three main contributions:

First, on the technical side (Section 2), we propose to instrument hash tables on the GPU to trace all occurring activation patterns in a data set and their statistics efficiently in realistic training scenarios. To the best of our knowledge, for the first time, this allows us to analyze complex scenarios such as the evolution of a deep residual network over the complete training process.

Second, on the experimental side (Section 3), we use this technique to inspect how architectural choices (CNN, CNN with expanding over-parametrization, ResNet, and PyramidNet), optimization methods (learning-rate schedules and FixUP), and hyperparameters (the network width) influence this evolution. All experiments were conducted on the CIFAR-10 data set (Krizhevsky, 2009), for efficiency and consistency. Third, our analysis provides answers to the following questions:

**How much information about the initialization is retained during training?**

We observe a general (although not complete) change of the activation patterns found in the initialization after only a few training steps. In contrast, ResNets maintain activation patterns in some layers from the initialization throughout the whole training process.

**Regarding the convergence of activation patterns in a network, where and when does structure form first?**

In all our experiments, the non-linear structure converges bottom-up (lower layers stabilize first). Noteworthy, both PyramidNet and ResNet with FixUp initialization, which provide the best results in our setup, show either a much more uniform convergence throughout all layers or a faster convergence in some network parts.

**How does the functional expressivity evolve during training?**

The distribution of activation patterns found in the data set in every deep network layer allows us to determine how much percent of the maximal possible expressivity a network uses for its decisions. For instance, if the distribution is heavy-tailed, the network uses much of its expressivity. If only a few activation patterns occur, the whole network could be approximated more easily using only one linear classifier.

Generally, we found that the expressivity increases with deeper layers in the initialization point. During training, the expressivity changes based on architectural choices:

• Residual networks remain in a high-expressivity state.
• Networks without residual connections lose that expressivity during training and slowly recover during further training (peaking again later in training).

Layer-wise, we also found architecture-based differences: ResNets converge to final layers of higher expressivity, while networks without residual connections converge to final layers of lower expressivity.

**Does the distribution of activation patterns confirm observations known from the literature regarding the early phase of training?** Through the lens of our framework, we can confirm the literature findings regarding the early phase of training and in accordance with the recent work on the early phase of training (Frankle et al., 2020); we also see a distinct behavior in the training dynamics during the first few optimization steps.

The remaining part of our article is structured as follows: In Section 2, we introduce our framework and provide the PyTorch (Paszke et al., 2019) code to retrieve the activation distributions efficiently and present novel measures that use these activation distributions. In Section 3, we use these measures to answer the questions given previously given typical training setups on the CIFAR-10 data set. Finally, we discuss our results and their relevance in the context of the related work in Section 4 and give a high-level explanation of the findings.

## 2 Materials and Methods

We aim to analyze the structural changes that occur during the training of deep networks with ReLU activations or general simple piecewise linear activations. To accomplish this, we examine, after each gradient descent step, all ever-occurring activation patterns of a network inside the whole training data set.

The intuition behind doing so is as follows: For simplicity, imagine a simple single layer inside a deep network that performs a linear affine transformation succeeded by ReLU activation, *x*↦max (*Mx* + *b*, 0). At any point in training, the layer (including the ReLU activation) can be described as a selection function

$$x\mapsto \left(x\mapsto M_{x}x+b_{x}\right),$$

that maps any layer input *x* to a fixed, but possibly distinct linear affine transformation (*M*_*x*_, *b*_*x*_) that acts on that input. This view allows us to get a qualitative understanding of the expressivity of that layer, as the total number of activation patterns used represents the number of linear transformations used to transform the incoming data. Thus, by counting the occurring activation patterns in the whole data set, we get a qualitative understanding of how “linear” the layer is.

The above intuition shows the qualitative feedback we get from measuring the *set* of activation patterns. In the following section, we define all measures used in this work; some that additionally take the distribution of activation patterns or two activation pattern sets at arbitrary training stages into account.

### 2.1 Notation

We analyze deep networks with piecewise linear activation functions *σ*(⋅) and *L* layers, denoted by $F:R^{d_{1}}\to R^{d_{L}}$ . Abstractly speaking, our analysis does restrict the type layers that can be used inside the deep networks, as we are only interested in the activation patterns in between all other layers. Thus, we can view all intermediate non-activation layers as black-box functions $f_{i}:R^{d_{i}}\to R^{d_{i+1}}$ . We decompose the whole network into layer blocks that do not contain any activation functions,

$$F_{l}≔\left(f_{l},\circ ,\sigma ,\circ ,f_{l-1},\circ \sigma \circ \ldots \circ \sigma \circ f_{1}\right)\left(x\right),$$ (1)

where ∘ denotes the composition operator, ***x*** denotes a data point of the data set, and the functions *f*_*i*_ denote the black-box layers inside the network that do not contain any non-linear activation function. Note that this notation implies *F = F*_*L*_. We define the *activation pattern on layer* *l* *of input* ***x*** as

$$a^{\left(l\right)}\left(x\right)≔\left(\delta_{>0}\circ F_{l}\right)\left(x\right)≔\left\{\begin{array}{cc} 1, & \text{ if }x>0\\ 0, & \text{else} \end{array}\right.$$ (2)

We define the *count of an activation pattern* ***a*** *on layer* *l* *under the input data set* Ω to be the number of its occurrences over the whole training set,

$$c^{\left(l\right)}\left(a\right)≔\left|\left\{x\;|\;a^{\left(l\right)}\left(x\right)=a,x\in \Omega \right\}\right|.$$ (3)

The total number of possible activation patterns on a particular layer *l* is $N_{l}≔2^{d_{l}}$ . Let $\left(a_{1},a_{2},\ldots ,a_{N_{l}}\right)$ be an arbitrary but fixed ordering of all possible activation patterns on layer *l*. Then, we define the occurrence vector of all activation patterns on layer *l* under the input data set Ω as

$$c^{\left(l\right)}≔\left(c^{\left(l\right)}\left(a_{1}\right),c^{\left(l\right)}\left(a_{2}\right),\ldots ,c^{\left(l\right)}\left(a_{N_{l}}\right)\right)\in N_{0}^{N_{l}}.$$ (4)

Last, we stretch the notation for ***c*** ^(*l*)^ at a particular network state after *s* training steps using ***c*** ^(*l*,*s*)^, which is the *occurrence vector at training step* *s* *on layer* *l*. For the vector ***c***, we denote the *i*th component using the notation ***c***_*i*_.

### 2.2 Measures

Using the notation of Section 2.1, we define the following measures on the discrete space of activation pattern counts:

**Cardinality of the Activation Pattern Set:** We start by defining the *total (distinct) pattern count*, defined as

$$\mathrm{total}\left(c\right)≔\underset{i}{\sum }\delta_{>0}\left(c_{i}\right).$$ (5)

As motivated before, the cardinality of the pattern set gives a broad view of how many distinct functions transform the incoming data. The measure is similar in spirit to the “number of regions” presented by Hanin and Rolnick, (2019b), but in contrast, our version takes the actual data distribution into account.

**Activation Pattern Changes:** Other work that focused on the early phase of neural network training measured the sign changes and magnitude changes of weights and their gradients (Frankle et al., 2020). Our framework does not take weights into account; instead, we measure the relative pattern changes that occur in between two training steps,

$$\mathrm{changes}\left(c^{\left(s\right)},c^{\left(s+1\right)}\right)≔\frac{\sum_{i}\delta_{>0}\left(c_{i}^{\left(s\right)}\right)\cdot \left(1-\delta_{>0}\left(c_{i}^{\left(s+1\right)}\right)\right)-\left(1-\delta_{>0}\left(c_{i}^{\left(s\right)}\right)\right)\cdot \delta_{>0}\left(c_{i}^{\left(s+1\right)}\right)}{\mathrm{total}\left(c\right)}.$$ (6)

In more detail, we measure how many patterns are present at step *s* + 1 but have not been present in the preceding step *s* and vice versa.

Furthermore, we analyze the distribution of activation patterns using two measures.

**Most frequent pattern count:** We measure the *frequency of the most frequent pattern*,

$$\mathrm{maxFreq}\left(c\right)≔\frac{\max_{i}c_{i}}{\sum_{i}c_{i}},$$ (7)

to gain a basic understanding of the proportion of a “default” case.

**Activation Pattern Entropy:** The most frequent pattern count, however, gives only a small insight into the typical inner workings of a network in case the activation patterns are equally distributed. Thus, we also define the *activation pattern entropy* as

$$H\left(c\right)≔-\underset{i}{\sum }\frac{c_{i}}{N_{l}}\cdot \log_{2}\frac{c_{i}}{N_{l}}.$$ (8)

This measure also gives a more detailed indication of how many functions the network uses to describe the data setup until a given layer, similar to the activation pattern set’s cardinality. Thus, if the mean activation entropy over all layers is very low, the whole network can be approximated with only a few linear maps. If the number is high, the network uses more of its non-linear capabilities. The difference to the cardinality measure is that entropy also tells us how equally the observed activation patterns are distributed.

**Relative Activation Entropy:** The activation pattern entropy does not yet give a comparable measure for different layers. For instance, a linear layer that uses *d* filters can use at most 2^*d*^ activation patterns, limiting the value for the activation pattern entropy to a maximum of *d*. However, the minimum activation pattern entropy is always 0 (if only one activation pattern occurs for every input). Consequently, one layer with few filters but equally distributed activation patterns can still yield smaller activation pattern entropy than another layer with many filters but a much skewed distribution.

To achieve comparability between layers and networks, we normalize the activation pattern entropy using the maximal activation pattern entropy of *d*. Additionally, the number of data points limits the number of activation patterns that can occur. For a layer with an output size of *w* ⋅ *h* pixels and a training data set consisting of *n* examples, the number of activation pattern evaluations is *n* ⋅ *w* ⋅ *h*. This also limits the entropy to a maximum value of log_2_ (*n* ⋅ *w* ⋅ *h*). Therefore, the maximal activation entropy for a layer with output dimension of *w* ⋅ *h* ⋅ *d* for a data set of *n* training examples is $\min\left(d,\log_{2}\left(n\cdot w\cdot h\right)\right)$ .

To summarize, we enable comparability between layers with different filters and output dimensions by defining the relative activation pattern entropy as

$$H_{r}\left(c\right)≔\frac{H\left(c\right)}{\min\left(d,\log_{2}\left(\sum_{i}c_{i}\right)\right)}.$$ (9)

**Measuring the Importance of the Initialization and the Convergence Rate of the Non-Linear Structures:** Last, we utilize the activation pattern distribution of two training states to analyze the importance of the initialization or the layer-wise convergence rate to the final training state. In more detail, we measure the number of shared activation patterns at two training states of a network. The idea is to compare if and to what extent two sets intersect, taking the value 0 if the two sets *X* and *Y* do not intersect and the value 1 if they are equal.

This could be done, for instance, by measuring the *Jaccard index* of the two pattern sets (without occurrence count), $JI\left(X,Y\right)=\frac{X\cap Y}{X\cup Y}$ . However, the Jaccard index does not fit our application as it does not take the numbers of the activation patterns into account. As a counter-example, consider using the Jaccard index to compare two experimental setups. Assume that the measurement of both setups results in the same number of distinct patterns, but one of the two experiments uses twice as many data points to estimate the change of pattern sets. The experiment using more data points would result in a lower Jaccard index. The reason is that the probability of a change is smaller if multiple data points were changed to let the Jaccard index notice a change in the pattern set.

To counter this, we use the *weighted Jaccard similarity*, also known as the *Ruzicka similarity*, instead as in:

$$J_{W}\left(c,c^{\prime }\right)≔\frac{\sum_{i}\min\left(c_{i},c_{i}^{\prime }\right)}{\sum_{i}\max\left(c_{i},c_{i}^{\prime }\right)}\in \left[0,1\right],$$ (10)

which takes the value 0 if the two occurrence vectors ***c*** and ***c*** ′ have no patterns in common and 1 if their occurrences are equal.

Our goal is to gain a better understanding of the patterns of the initialization and the convergence rate of activation patterns inside a network. Thus, we define two measures using the weighted Jaccard similarity: First, we measure $J_{W}\left(c^{l,\cdot },c^{l,s_{\text{init}}}\right)$ that estimates the number of patterns from initialization that is still present during any other point in training.

Second, we also measure when the training lets the activation patterns reach their final activation pattern state, $J_{W}\left(c^{l,\cdot },c^{l,s_{\text{final}}}\right)$ . This measure estimates the convergence rate at which the non-linear part of the network reaches its final structure. We accomplish this by training the network, saving all intermediate network states, and comparing every saved state with the activation patterns of the final network state.

### 2.3 Efficient Activation Pattern Counting

For practical use, defining a list of all possible activation pattern counts in a realistic setting is not feasible. For instance, 16 filters would require to reserve only about 2 GB of memory on the system, but 32 filters would require 137 TB of memory. The alternative would be to use a dictionary, as the number of data points in the data set limits the number of measured activation patterns. This technique, however, would slow down the measurement considerably, as we would have to either implement a dictionary lookup directly on the GPU or copy the patterns of the processed data to the host machine during training. Instead of listing all possible patterns, we define hash lists of fixed size for every layer and fill them according to Algorithm 1, which queries a hash list of activation pattern counts efficiently on the GPU. We also include the occupancy of the hash list in our analysis in Section 3 and use it to discuss the validity of all other measurements.

**Algorithm 1:** count_act_ This function uses a hash table to quickly count the occurrences of all unique activation patterns.

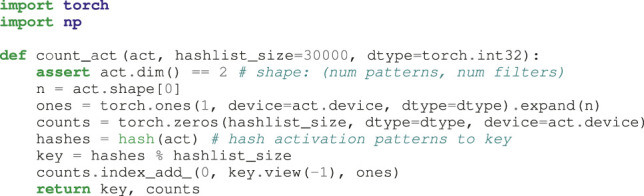

## 3 Results

In the following, we analyze the training of typical convolutional neural networks through the lens of our framework. The experiments and observations not only aim to extend the work of Frankle et al. (2020) and Hanin and Rolnick (2019b) on the early phase of neural network training but also to give new insights, enabled by the novel view of observing the structure of the non-linear part of a deep network only.

In all our experiments, network training is non-invasive; with every batch-wise training step, we freeze the model and measure all activation patterns that occur on a per-layer basis when feeding the network with the complete training data set. We measure the activation patterns according to formula Eq. 3 for every step in training. If a network contains batch normalization layers, we set these layers to *training mode* (i.e., using the actual mean and variance of the training batches for normalization) to mimic the internal structures for all input images that would arise during training.

### 3.1 Experimental Setup

In the following, we examine the training process of four different networks. To maintain comparability, we use the CIFAR-10 (Krizhevsky, 2009) data set for all of our experiments.

As a baseline, we used the ResNet-20 in its v2-CIFAR variant as described by He et al. (2016b). We analyze the choice of three architectural features used in the ResNet architecture. First, we examine the exact effect of the skip (also known as *identity*) connections. In more detail, we define ConvNet-20 to have the same structure as the ResNet-20 but with the skip connections removed. Second, we define ToyNet-20, a ResNet-20 variant, without any skip connections and an equal filter count (32 filters) for each convolutional layer in the network. In contrast, ResNet-20 uses increasing filter counts for every block (16, 32, and 64 filters). Last, for some experiments, we additionally include a PyramidNet (Han et al., 2017) variant, PyramidNet-20, of ResNet-20 that uses a linear increase of filters in every successive layer.

All networks have an additional average pooling and a classification layer at the end. These do not appear in the following analysis, as these are not preceding an activation layer—our framework only considers the non-linear structures given by discrete activation patterns inside a deep network.

As we have used hash lists to measure the complete set of activation patterns and their frequencies, we also adjust the occupancy of the hash lists. We adjust the hash list size to use the maximal capacity of 11.6-GB GPU-RAM to fit the GPU memory. To maintain a low occupancy for layers that undergo many distinct patterns and maintain comparable results across all layers, we balance the hash map sizes across layers adaptively to have maximal occupancy 15% of the size of the hash list.

The used hyperparameters, the exact model definitions, and method implementations can be obtained from the provided code under https://github.com/JGU-VC/activation-pattern-analysis.

### 3.2 Observations in the Early Phase of Training

Previous works have already identified the early phase of training to have training dynamics distinct from the rest of the training process (Section 4.1 on the related work). In the following, we validate the observations from the literature on the early phase of training: these include rapid sign changes, converging gradient magnitudes, the alignment of momentum that indicates a more linear rather than non-linear learning phase, and the finding that corrupts data (bad batches) might cause irrevocable damage. Additionally, we analyze how much of the structure of neural networks given by its random initialization is maintained throughout training.

**FIGURE 1:**
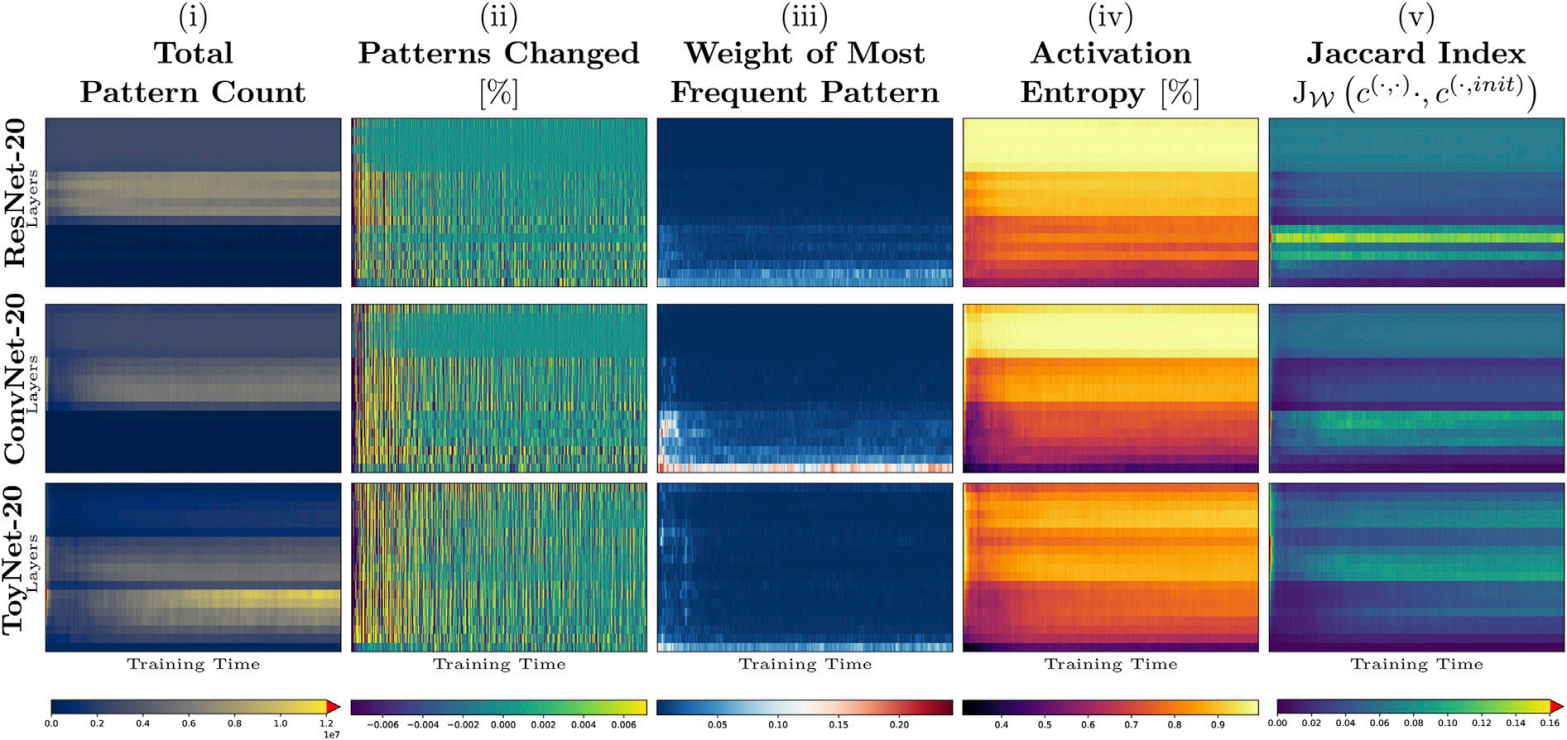
Early training phase of ToyNet-20, ConvNet-20, and ResNet-20 (first 3,000 iterations) on CIFAR-10. The top row represents the last convolutional layer before the linear classification layer. The bottom row corresponds to the first convolutional layer in each image. The images, from left to right, show: **(A)** the size of the activation pattern set, relative to the maximum size seen during training; **(B)** the change of size of this pattern set, truncated for better visibility; **(C)** the relative frequency of the most frequent activation pattern; **(D)** the activation entropy; and **(E)** the weighted Jaccard similarity comparing the current activation patterns to those directly after network initialization.

Our first experiment tracks the activation pattern distributions during the first 3,000 iterations of the training process on CIFAR-10 using the ResNet-20, ConvNet-20, and ToyNet-20 architectures. (In each case, we use the same hyperparameters optimized for ResNet-20 that achieve a test accuracy of about 91*%* without additional data). In each training step and layer, we evaluate the total number of patterns, the number of pattern changes, the weight of the most frequent pattern, the activation entropy, and the weighted Jaccard similarity to the pattern occurrences at the initialization of the network. We show the results for each measure in Figure 1, using the whole training data set for evaluation.

In Figure 1A, we observe that the *absolute number of patterns per layer* differs by several orders of magnitude for each architecture. While the number of patterns ranges from maximally 22 million patterns in the first convolutional block to about two million patterns in the last block of the ToyNet in the initialization state of the network, the number of distinct patterns drops significantly after the first step to maximally three million patterns. The number of patterns remains constant in the last convolutional block. The ConvNet-20 and ResNet-20 models behave differently: the first blocks in both models have the fewest distinct activation patterns, and the middle blocks have the most activation patterns in the later stages of training. By measuring the *total (distinct) pattern count*, we validate the work of Hanin and Rolnick (2019b), who have shown that ReLU activations are rather sparsely distributed. We validate their observation that the number of “activation regions” (or activation patterns in our notation) first drops after initialization and increases slowly with training again.

Figure 1B shows how many pattern changes occur at each layer for each training step. In accordance with related work that analyzed sign changes of weights (Frankle et al., 2020), most pattern changes occur during the first few steps in all our experiments.

Interestingly, during training, pattern changes may strike from the lower to the top layers simultaneously. As new patterns first appear and then the same amount of patterns disappear again or vice versa, this might be evidence for unfavorable batches for training. We observed that these strikes appear in all networks. For ToyNet, we see that most activation patterns disappear from the pattern sets in the first training steps. The ResNet, in contrast, shows a more stable distribution over pattern set changes. We believe this potentially gives a hint why ResNet architectures are easier to train. We will see later why ResNets might not require many structural changes.

Figure 1C shows the weight of the most frequent pattern per layer and training step, observed in the whole data set. A high-density pattern indicates a non-uniform distribution of activation patterns. The most frequent patterns arise for the ToyNet variant, for example, only in the first hundred training iterations or the first activation layer. In contrast, the ResNet network has the fewest high-density patterns in the first few hundred steps during training but maintains higher densities for the first few layers during the whole 3,000 steps. Remarkably, for the ConvNet variant, some layers have patterns that account for up to 25*%* of all observations, indicating possibly unnecessary structures inside the network. However, we observe these high-density patterns only intermediately—after a few hundred training iterations—the most observed patterns in all layers, except for the first layer, only account for less than 2*%* of all pattern evaluations. They even account for less than 0.001*%* of all pattern evaluations in deeper layers, giving potential new insights for online pruning methods that take combinatorial frequencies of activations into account.

Next, we analyze if and to what extent the activation patterns of the initialization help with the training. The goal is to determine what kind of architectural features utilizes the initialization structure instead of using it only as a starting point and changing its nature completely. Thus, we consider the weighted Jaccard similarity between the pattern occurrence of the initialization ***c*** ^(*l*,0)^ and the current training step as shown in Figure 1E. In the first few steps, the similarity to the initialization is unsurprisingly the highest. It then drops to 0% in the worst case and 16% in the best case. The ToyNet-20 model has the fewest overlap of activation patterns to the initialization as a general statement among the other two analyzed models. For this architecture, the similarity first drops to about 1–5%, and during training, both numbers increase to 4–10%. This indicates a rediscovery of some patterns that have already been presented at the initialization. Despite having a similar architecture, ConvNet-20 and ResNet-20 behave differently than the ToyNet-20 model. The most significant overlap of the “current” pattern set with that of the initialization appears in the bottom layers containing only 16 filters. This is probably because those patterns are more universal (edge or color detectors) and thus easier to sample (Zeiler and Fergus, 2014). The ResNet-20 shows a more considerable overlap of the patterns during training to the set of the initialization state than ConvNet-20, especially in the lowest block.

### 3.3 Architecture-Specific Entropy Curves

**FIGURE 2:**
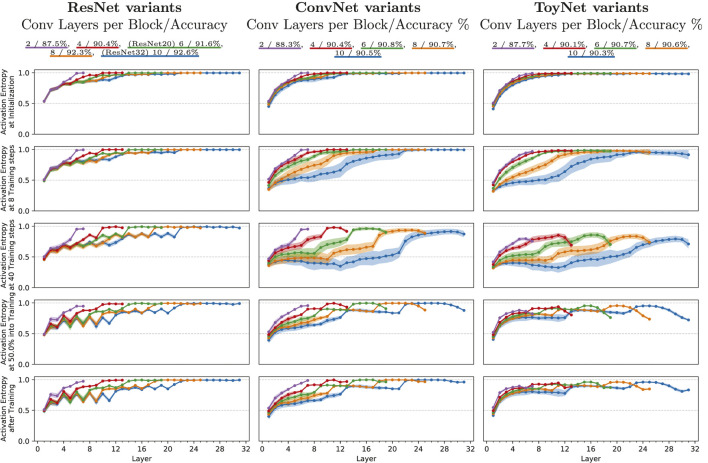
Mean relative activation entropy for ResNets and ConvNets of various depth, measured for an ensemble of 25 training runs. The measurements were taken separately for each layer at different points of the training process: (i) at initialization; (ii) after 40 steps of stochastic gradient descent; (iv) after four epochs; and (iv) right after the first learning rate drop at epoch 101. The shaded area indicates the variance spanning one standard deviation in each direction. The ResNet variants use the architecture proposed for CIFAR-10 by He et al. (2016a) and pre-activation shortcuts (He et al., 2016b), with a varying number of convolutional layers at each feature map size, shown in different colors. The ConvNets use the same basic architecture but without the residual shortcuts.

Our second experiment, shown in Figure 2, analyzes the evolution of the activation entropy throughout the training process. This experiment aims to relate the respective layers in each architecture to the expressivity emerging at a particular moment in training time. The relative activation pattern entropy rates the expressivity of an activation layer. A low expressivity layer (i.e., having low activation pattern entropy) could be more easily approximated by one single linear layer. In contrast, a high expressivity layer (i.e., having high activation pattern entropy) behaves more like a hash table in the sense that it has sufficient expressive power to memorize the output for many input data.

We specifically analyze the *mean* relative activation entropy (ActEnt) of 25 training runs of each network architectural change. The color represents the networks’ depth and ranges from 2 to 10 convolutional layers per convolutional block. To analyze the effect of architectural change, such as skip connections, we repeat the same experiment using ResNet-20, ConvNet-20 (ResNet-20 without skip connections), and ToyNet-20 (ResNet-20 without skip connections and constant filter sizes). Figure 2 shows the mean relative activation entropy (y-axis) for each architectural change for each layer (x-axis). The columns represent the three ResNet variants: ResNet-20, ConvNet-20, and ToyNet-20. Each row corresponds to a specific point in training: 1) the initialization state of the networks, 2) after eight training iterations, 3) after 40 training iterations, still at the beginning of the early phase of training, 4) at 50% into training, right before the learning rate drops due to the multi-step learning rate scheduler, and 5) after training. The equally colored hose around the graphs indicates the single standard deviation of the 25 measurements using different random seeds.

We first examine the initial state of training. For all network variants, the relative activation pattern entropy increases with deeper layers. Its value ranges from about 0.5 (half of the maximal possible expressivity) to about 1 (the maximal expressivity for the respective layers).

The ToyNet variant has the most evident increase of ActEnt; the ConvNet runs into saturation at the end of the second block, which only gets a new increase within the next block. After only a few steps of training, the ActEnt decreases for all network types, most for the ToyNet variants and least for the ResNet variants.

At step 40 of training (third row), the ActEnt further drops for all network types, specifically for ConvNet and ToyNet. The measurements for those two networks have the most significant standard deviation, especially deeper networks have a more extensive spread in their measurements than shallower nets. From the initialization point to this point in training, a characteristic structure is increasingly forming for the ResNet variant. Specifically, the ResNet variant exhibits a defined zigzag pattern in the entropy value, where layers before skip connections have lower ActEnt than layers after skip connections. Globally, the ActEnt is, however, still increasing.

In the final state of training, the standard deviation of activation entropy decreased again to similar values to those of the initialization phase. The zigzag pattern of the ResNet variants has strengthened slowly in the first block, while the last block is saturated to the maximal possible activation entropy again. The ConvNet variants and in particular, the ToyNet variants show a saturating increase of ActEnt in the first block, a slight decrease of ActEnt in the second block, and a decrease of ActEnt in the last block; the first layer of every new block shows a steep increase of ActEnt.

To conclude, this finding suggests that the ResNet architecture maintains its expressivity throughout training. In contrast, the missing skip connection results in the loss of expressivity in the first few training steps. Successful training, however, regains this expressivity slowly. The second feature of ResNet, the increasing number of filters, results in high-expressive final layers. In the case of ToyNet that does not include increasing filter sizes, the final layers decrease throughout training. However, this might be important for the cascaded nature of deep networks, combining more complex features until, in the final layers combinatorical-wise, many results have to be covered to get a good performance. We will discuss this further in Section 4.

### 3.4 Observations of State-Of-The-Art Architectures and Methods

**FIGURE 3:**
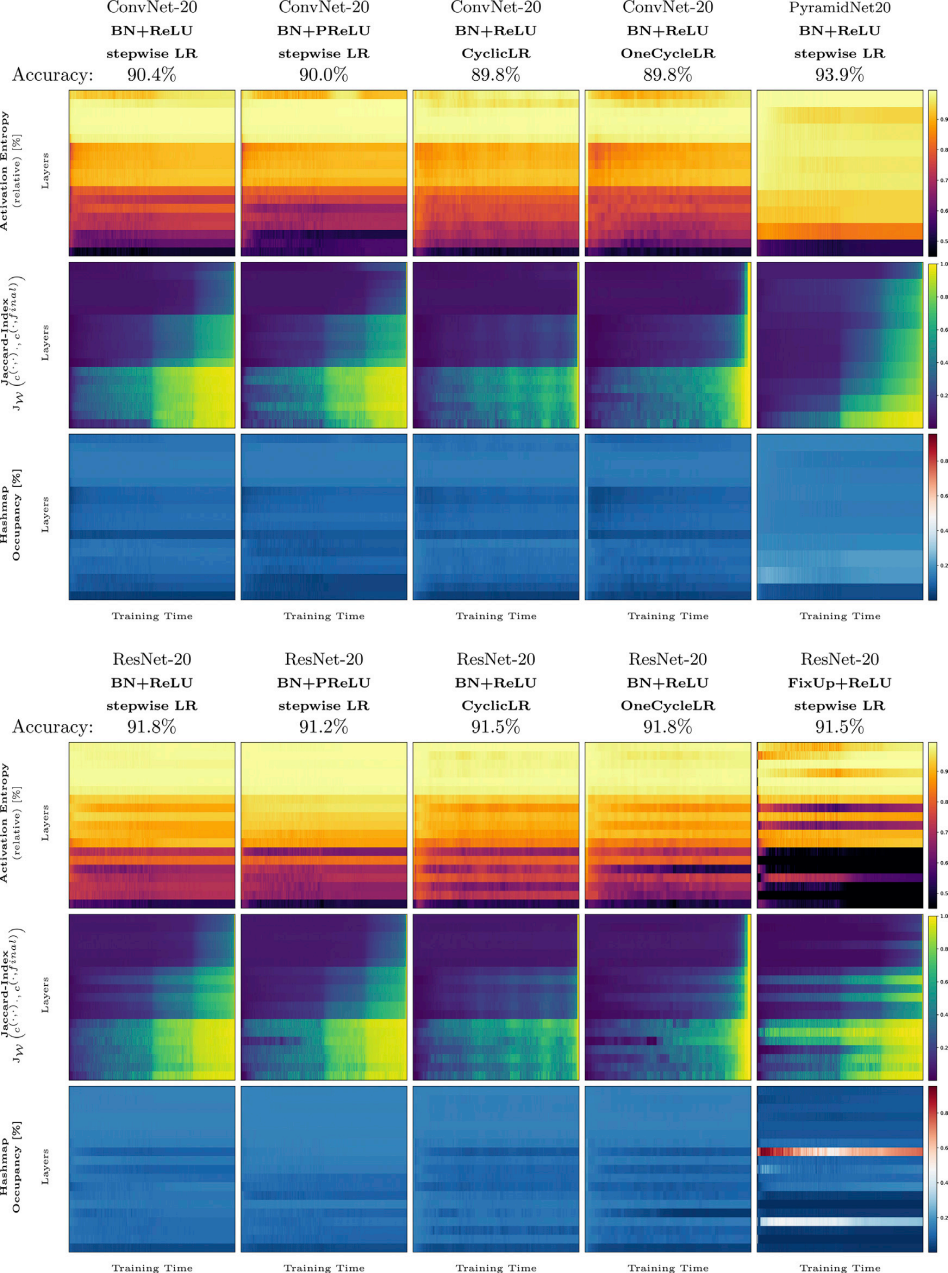
Complete training runs for several methods and architectures: ConvNet-20, ResNet-20, PyramidNet-20, FixUp, cyclic, and one-cycle learning rate schedulers. Each plot shows a measure based on activation pattern distributions throughout the whole training (x-axis) for all activation layers in each network (y-axis). The first row shows the activation entropy, the second row shows the Jaccard index between the current network state during training and the final network state, and the third row shows the hash list occupancy used to compute the distributions.

Our third experiment (Figure 3) applies two of our measures to the whole training procedure of several methods that are known to improve the training of image classification. In detail, we measure the effect of ReLU (Nair and Hinton, 2010), PReLU (He et al., 2015), cyclic learning rate scheduler (multiple and one cycle, Smith (2017)) on ResNet-20 and ConvNet-20, PyramidNet and FixUp (Zhang et al., 2019) on the measures ActEnt, and the Jaccard similarity of the current training step to the final network state, $J_{W}\left(c^{\left(\cdot ,\cdot \right)},c^{\left(\cdot ,\text{final}\right.}\right)$ . The first measure, ActEnt, gives a rationale of how much of their non-linear capabilities the networks use per layer. The second measure, the Jaccard similarity to the final network state, represents the activation-wise convergence of the networks on a per-layer basis. We include the layer-wise hash list occupancy for all setups for the whole training procedure to validate our measurements. As described by Hanin and Rolnick (2019b), activation patterns appear only sparsely. To maintain comparability, we adapted PyramidNet and FixUp to use 20 layers as well. Note that this results in fewer activation layers for PyramidNet and thus fewer rows, as our framework targets activation layers exclusively. For the setups labeled “stepwise LR,” we use a stepwise learning rate scheduler, reducing the learning rate after precisely the first half of the training by a factor of 10, and then again by a factor of 10 at the start of the last quarter of the training.

First, we describe the general behavior of ActEnt for all experiment setups. Generally, the ActEnt increases with deeper networks. In the initialization state of training, ActEnt achieves greater values (as described in Section 3.3) but as described in the previous section, drops for some layers after only a few steps of training. The layers that show a reduction in entropy in the early training phase slowly regenerate entropy as the training proceeds. With the reduction of the learning rate, the activation entropy in the last layer gets a new boost and briefly rises again, except for PyramidNet-20, discussed later in more detail.

Remarkably, the cyclic learning rate schedulers, “CyclicLR” and “OneCycleLR,” dampen the effect of activation entropy drop in the early phase of training. Nevertheless, the initial decrease of activation entropy still occurs in the middle layers of the networks. Both methods also result in lower ActEnt in the last three layers. For the ResNet-20 setup, where “OneCycleLR” is probably used most often, the method increases ActEnt in the first convolutional block the most among all other methods. From the named methods, “PReLU” dampens the drop of activation entropy the most in the middle and the third convolutional block of the networks for ResNet-20. It shows a more significant reduction in entropy in the first block and for ConvNet-20 as well in the last convolutional layer. The “PReLU” method also inverts the zigzag pattern (previously described in Section 3.2) for ResNet-20 in the middle convolutional block. FixUp, which (in contrast to the other approaches) works without batch normalization layers, shows a distinct behavior: except for only a few layers in the middle, the first and third convolutional blocks have the smallest value of ActEnt among other methods throughout training. In contrast, PyramidNet-20, having a linear increase in filters, has the most gradually increasing and, over time, most stable entropy curve: its value increases quickly, starting with a low ActEnt value at the beginning of training. Only two activation layers (6 and 9) lose ActEnt over training time. Despite their completely different behavior compared to the other methods, FixUp and PyramidNet have high accuracy.

Next, we analyze the methods based on the Jaccard similarity to the final network state. The measure gives a rationale for the convergence of the activation patterns. In the default case (ConvNet-20 with ReLU activation), all layers start with low similarity compared to the final network state. After just a few epochs, the activations in the first convolutional block have a similarity of about 50*%*. This means that about 50*%* of all patterns (weighted by their occurrence count) are already exactly those of a trained network. Until the first learning rate reduction at epoch 100, the second block reaches a similarity of about 17*%*. The last layer does not increase significantly during that phase, starting at about 5–6*%*, the layer reaches a similarity of 6–7*%*. With a decrease of the learning rate at epoch 100, the similarity increases within a few steps to about 97*%* in the first block, about 60*%* in the second block, and about 30*%* in the last block. In the very last few epochs, all layers converge to a similarity of 100*%*. The other training setups differ in the following: All ResNets have a zigzag pattern, just like in the activation entropy plots. Compared to entropy, however, the high and low points differ from method to method; while the similarity of the middle block reaches its maximum for “ResNet-20, BN + PReLU, stepwise LR” in the layers that directly succeed a skip connection, the similarity of “ConvNet-20, BN + PReLU, stepwise LR” is flipped across layers after or before skip connections. Despite having no skip connections, the plot for the setup named “ConvNet-20, BN + PReLU, stepwise LR” shows a zigzag pattern in the similarity, as does the ResNet-20 variant with PReLU. The training runs that use a cyclic scheduling exhibit a wave-like pattern over the training period, having a higher value whenever the learning rate is lower and vice-versa. The single training runs that use the cycle learning rate scheduler, denoted by “OneCycleLR”, start to converge very late compared to the other methods. However, these runs also show the most uniform convergence across all layers and converge faster than the other methods. The Jaccard similarity during the whole training procedure of “PyramidNet20” reveals that the architecture has the most uniform increase of the Jaccard similarity, both layer-wise and training time-wise. The first layer has the more similar pattern sets throughout training than the final network states’ pattern set. The similarity of the subsequent layers behaves similarly but with a lower similarity value. Only the last layer does not converge as quickly as the other layers. FixUp is probably the most distinct in terms of the behavior of the Jaccard similarity (as it is also for activation entropy). FixUp manages to reach some of the final activation patterns for some layers (5, 6, 7, 10, 12, and 16) much earlier in training than the other methods. The remaining layers have approximately the same course as the other default training setup, “ResNet-20, ReLU, stepwise LR”.

## 4 Discussion

Stochastic gradient descent (SGD) (Robbins and Monro, 1951) does not directly optimize the activation patterns themselves—it still remains unclear why and how the internal structure of neural networks appears during training. On a small scale, a training step weight update either changes a data point in such a way that it crosses a ReLU (Nair and Hinton, 2010) decision boundary hyperplane, or the weight update only improves the regression of the decision in the final layer. Which case occurs is somewhat random and depends on factors such as the learning rate, the used optimizer, and the exact data distribution, to name a few. In this section, we will review the results described in the last section and connect them to the literature, shedding some light on activation pattern distributions that arise with the choice of architectures and methods. We will conclude by answering the questions we asked at the beginning of this work.

### 4.1 Related Work

The topics discussed in our work are tightly bound to recent findings in the literature: From a broader point of view, optimization has been an active field of research for decades in the context of neural networks (see Schneider and Kirkpatrick (2006) for general introduction and Goodfellow et al. (2016); Montavon et al. (2012) for an introductory text in the context of neural networks, specifically stochastic gradient descent (SGD) (Robbins and Monro, 1951)), especially recent works have pushed state-of-the-art performance by evolving specific niches of optimization techniques such as faster converging learning rate schedules, better network initializations, or activation functions: Many of these methods that improve the quality of a particular set of data or a particular architecture originate from intuitive approaches. For instance, Smith and Topin (2017) and Smith (2017) found an analogy of LR schedules to simulated annealing. The research in different activation functions has also been vast; the most commonly used piecewise linear activation functions for image classification today are ReLU (Nair and Hinton, 2010) and PReLU (He et al., 2015). Architecture-wise, plain convolutional neural networks have been replaced by ResNet variations (He et al., 2016b; Szegedy et al., 2016). Also, several research articles have already focused on why skip connections result in higher quality networks (Balduzzi et al., 2017; Orhan and Pitkow, 2018). Two slightly more recent variants of ResNet are PyramidNet (Han et al., 2017), which uses a gradually increasing number of filters, and FixUp (Zhang et al., 2019), which optimizes the different weight types in a network separately and changes the initialization scheme. Our work compares several of the named architectural decisions in terms of activation pattern entropy and activation pattern convergence.

Our work is closely related to the ongoing discussion about the distinct phases of training (Achille et al., 2019; Frankle et al., 2020; Leclerc and Madry, 2020). Our work extends this by analyzing the complete training process and investigating multiple architectural choices such as activations, ResNet variants, and learning rate schedulers.

Most related to this work in terms of interpreting network activations to probe learning dynamics are Raghu et al. (2017) and Gotmare et al. (2019). In these works, the authors propose a new technique for comparing network representations using singular value decomposition and canonical correlation analysis but do not use statistical measures. Other works focusing on the analysis of the weights of a network are inherently limited, since the weights exhibit several invariances, such as permutation and scaling, as Gotmare et al. (2019) note.

In contrast to the named studies, we focus on the discrete activation patterns rather than the continuous view of values, weights, gradients, and the similar.

Several recent articles discuss the use of statistics to guide optimization methods (Lang et al., 2019; Xu et al., 2019) but only use the training and validation losses to approximate the training dynamics. In Moldovan et al. (2020), the authors use the so-called transfer entropy between network nodes to guide backpropagation. Other works use statistics for feature extraction (Finnegan and Song, 2017), feature pooling (Wan et al., 2019), or network compression (Wiedemann et al., 2019). Related to that is the research on the distribution of activations, which often treats all neurons as independent stochastic variables and has proven helpful for derivations of initialization schemes and methods to help with training (Glorot and Bengio, 2010; He et al., 2015; Ioffe and Szegedy, 2015; Salimans and Kingma, 2016).

In contrast to these works, our emphasis lies on the study of the discrete distribution of layer-wise activation patterns in a deep neural network, explicitly analyzing their internal non-linear structures.

The closest related work that also takes activation patterns into account is probably that of Hanin and Rolnick (2019a) and Hanin and Rolnick (2019b), where the authors analyze the capabilities of neural networks in terms of expressivity. They show that neural networks use much fewer activation patterns during training than theoretically possible. In contrast to our work, they did not consider the input distribution but analyzed the whole theoretical input space. We validate their results in terms of actual occurring and observed activation patterns during training. Additionally, we provide observations regarding the dynamics of training.

### 4.2 Methodical Considerations and General Findings

The activation pattern entropy reveals the internal complexity of a neural network throughout training. This measure indicates qualitatively how many distinct linear transformations a deep network uses for inference. If a network uses only a few distinct linear transformations (activation patterns), this corresponds to a regression task that just blends between data. On the other hand, a network that uses many distinct linear transformations comes close to have such a mapping for every element in the data set.

Our experiments (e.g., Figure 3) reveal the extent of expressivity throughout training, and they show that this depends on the architectural choices used for the networks. For instance, the ResNet architecture and skip connections in particular, indicate an alternating scheme, using fewer patterns (i.e., being more linear) in layers that succeed the skip connections directly and using more patterns in each other layers. Removing these architectural choices (such as skip connections) and increasing filter sizes (ToyNet) result in activation entropy valleys during the early and later training phases, meaning sudden losses of expressivity. Methods that reduce training speed (PReLU, 1-cycle learning rate scheduler, and cyclic learning rate scheduler) may counter this effect again.

### 4.3 Initialization (and the First Steps of Training)

The most common method of initializing deep neural networks that use ReLU activations today is He et al. (2015). Its idea is that a very precisely scaled Gaussian distribution results in i. i.d. random activations per neuron, that is, maximally entropic activation patterns. In theory, the condition of independent input dimensions may be correct for specifically designed data sets. However, the independence of input dimensions is not met by real-world data (for instance, proximate pixels in photographs correlate) and cannot be correct for deeper layers (as the inputs have been mixed in the previous layers already). In support of this, one can name the numerous works that have developed successful methods for training even without this assumption, for example, the exponential learning rate scheduler (Li and Arora, 2020), super-convergence (Smith and Topin, 2017), and fixed-update initialization (Zhang et al., 2019). The strict increase of activation entropy in the initialization phase (see Section 3.3) can be seen as a validation that the possibly unfulfilled condition “i.i.d.” used to prove He-initialization does not impact the initialization negatively. Previous works have given many reasons why the ResNet architecture is beneficial to training (Balduzzi et al., 2017; Orhan and Pitkow, 2018). Our measures give a new indicator why this might be the case.

**FIGURE 4:**
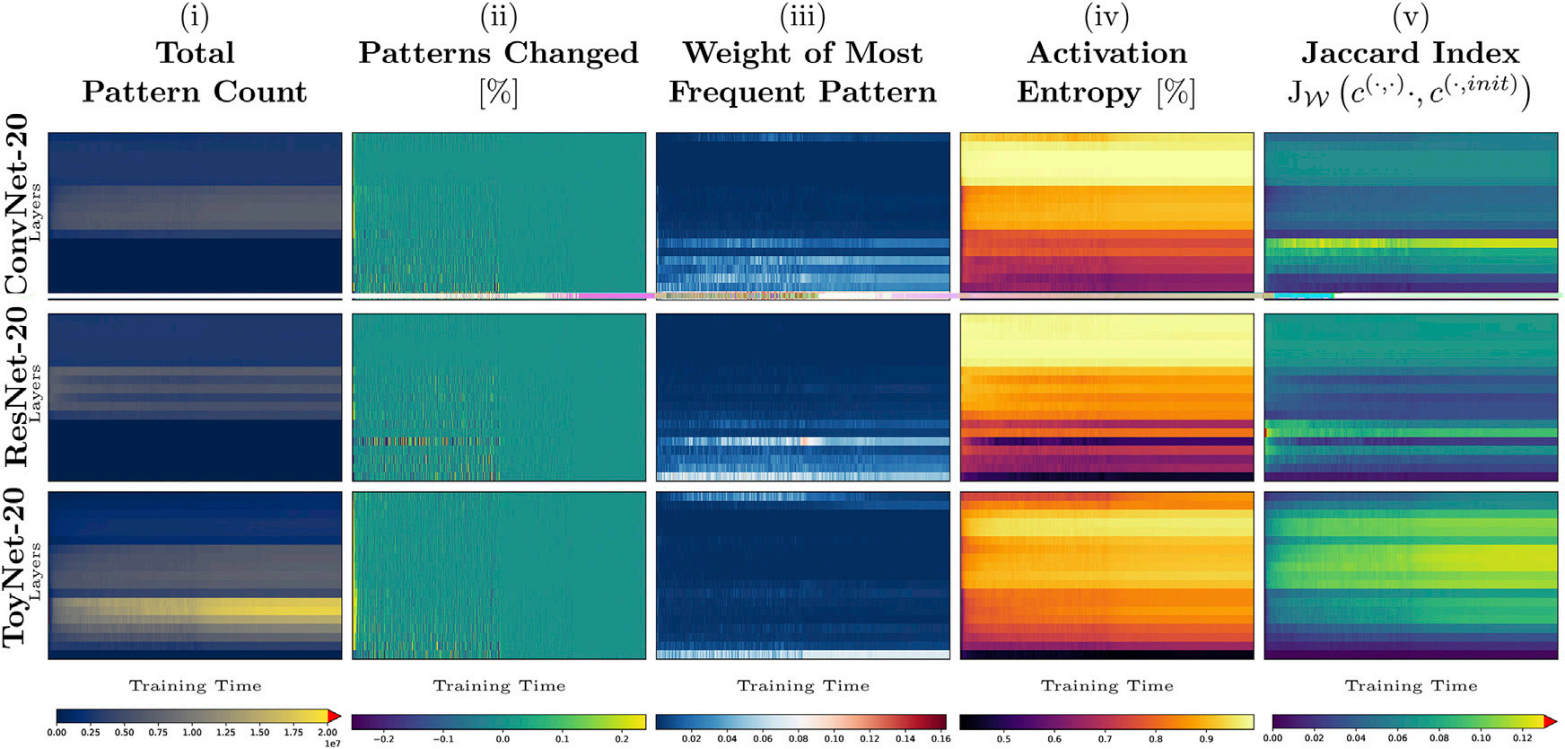
Full training phase (of 200 epochs) of ToyNet-20 **(A)**, ConvNet-20 **(B)**, and ResNet-20 **(C)**. The plots correspond to those in Figure 1, showing the measures from left to right: total pattern count, patterns changed, weight of most frequent pattern, the activation entropy, and the Jaccard index between the current network state and the initial network state.

In Figure 4, we have measured the Jaccard similarity to the initialization state over the entire training time. The two models, ConvNet-20 and ToyNet-20, lose most activation patterns of the initialization but find some of the patterns again during the training process. In contrast, the ResNet architecture shows a less steep decrease, and in some layers, the architecture manages to maintain some activation patterns of initialization over extended periods in training. Our analysis, however, does not cover whether the activation patterns of initialization are meaningful for the network’s performance, and thus this remains an open question for future work.

### 4.4 The Early Phase of Training

The early phase of training has been identified in several works to be distinct from the rest of training. Goodfellow and Vinyals (2015) described that gradient magnitudes are substantial during the first *˜*10 steps of training. In our work, we found that during that phase, the activation entropy drops in almost all layers (Figure 2 and Figure 1). A similar reduction of expressivity and a subsequent recovery phase of activation entropy have also been previously observed by Hanin and Rolnick (2019b) in terms of the theoretical total number of activations over the whole input domain. We validate that this theoretical change in activations is also present for more realistic setups (architectures and methods) and the actual data fed into the networks.

Frankle et al. (2020) describes the first 500 steps of training to undergo a substantial “rapid motion” in weight space. Our measurements reveal similar observations: the pattern changes, especially in the middle layers decrease, the most frequent patterns even out in the lower layers, and the activation entropy increases again (Figure 1).

In the next training phase, described by Gur-Ari et al. (2018) to end at training step 700, the Hessian eigenspectrum separates; the gradient lives in a much smaller subspace than before. Also, in that phase, the direction of the momentum starts to align (for VGG-networks). In our study, the magnitude of pattern changes converges for some layers, and the activation entropy stabilizes. Also, that phase had the most similar activation patterns to the initialization state of the tested networks.

After that (starting at about training step 2000 in our setup), Frankle et al. (2020) characterize training to have a constant magnitude spectrum of gradients and slow increase of weight magnitudes. Our measures reflect these observations as the magnitudes of pattern changes converge in the whole network to a hitherto minimum value.

The pattern changes also reveal which batches change the structure the most. We have described sudden strikes of pattern changes in Figure 1; first, removing many patterns in all layers at the same time and then adding patterns back in an equal amount. Achille et al. (2019) have shown that unfavorable data early in training can corrupt the whole training process resulting in irreparable damage to the network state. We argue that the measure of pattern changes can help identify such counter-productive data points or batches.

### 4.5 Convergence Speeds of Methods

In Figure 4, we have pre-computed the network training to observe the convergence rate of activation patterns throughout training. Our findings indicate that networks converge first in the lower layers and from there, bottom to top. More specifically, all but the first layers do not converge slowly. For instance, the similarity to the final network state reaches a similarity of about 30*%*, meaning that the remaining patterns are still not converged. We assume that especially the activation patterns of deeper layers are very noisy, as the similarity only converges in the last few steps. The best behavior in terms of the fastest convergence has PyramidNet and FixUp. While the former manages that all layers converge almost at the same pace, the latter ensures that some layers converge very early in training.

## 5 Conclusion

Non-linearities are the main reason for the high expressivity of deep networks. As a prominent example, the ReLU non-linearity uses a simple decision boundary to break the linear flow of calculations. Surprisingly, although optimization using stochastic gradient descent does not take that decision boundary directly into account, the decisions still may change during training due to overshooting their boundaries upon some gradient descent steps. This work aims to quantize these discrete changes, reinterpreting the continuous optimization as a discrete search of emerging structures. In more detail, this work analyzes where, when, and how quickly such structures arise during training. Unsurprisingly, given the magnitude of the problem, our experimental analysis does not provide an overall model of the implicit algorithm of discrete optimization. The value and main contribution are to connect common architectural and training approaches with the resulting change of discrete optimization.

In order to gain an understanding of the interplay between the emergent discrete–continuous network structure and continuous optimization, we analyzed which layers learn first in a network and found that the answer is much more diverse than just the first or the last layer. In more detail, our experiments show that ResNets do not throw the patterns of initialization away during training, in contrast to similar architectures without skip connections, which replace the pattern set of the initialization almost entirely within only few steps of training. We used our measures to validate other effects observed in the literature, for instance, Frankle et al. (2020); Leclerc and Madry (2020); Hanin and Rolnick (2019b); and Achille et al. (2019). We have shown that a network’s expressivity (or activation pattern entropy) undergoes an architecture- and training method-specific curve during training, often dropping after only the first few training steps. We have tested this behavior against several methods, such as PReLU, ResNet skip connections, some learning rate schedulers, and PyramidNet or FixUp, which particularly counter that effect and may maintain higher expressivity throughout training or boost the convergence of some network parts.

We believe that the provided analysis of the non-linear convergence of neural networks, their expressivity used for training, and the importance of initialization could give new tools to find more efficient training methods or architectures and potentially enables new avenues toward understanding the optimization of neural networks in general.

## Data Availability

Publicly available datasets were analyzed in this study. These data can be found at https://www.cs.toronto.edu/∼kriz/cifar.html.
